# Factors associated with high-level endurance performance: An expert consensus derived via the Delphi technique

**DOI:** 10.1371/journal.pone.0279492

**Published:** 2022-12-27

**Authors:** Magdalena J. Konopka, Maurice P. Zeegers, Paul A. Solberg, Louis Delhaije, Romain Meeusen, Geert Ruigrok, Gerard Rietjens, Billy Sperlich

**Affiliations:** 1 Care and Public Health Research Institute, Maastricht University, Maastricht, Limburg, Netherlands; 2 Department of Epidemiology, Maastricht University Medical Centre, Maastricht, Limburg, Netherlands; 3 School of Nutrition and Translational Research in Metabolism, Maastricht University, Maastricht, Limburg, Netherlands; 4 Norwegian Olympic and Paralympic Committee and Confederation of Sports, Oslo, Norway; 5 Human Physiology and Sports Physiotherapy Research Group, Vrije Universiteit Brussel, Brussels, Brussels-Capital Region, Belgium; 6 Brussels Human Robotics Research Center (BruBotics), Vrije Universiteit Brussel, Brussels, Brussels-Capital Region, Belgium; 7 Integrative & Experimental Exercise Science & Training, Institute of Sport Science, University of Würzburg, Bavaria, Germany; Universidade Federal de Mato Grosso do Sul, BRAZIL

## Abstract

There is little agreement on the factors influencing endurance performance. Endurance performance often is described by surrogate variables such as maximum oxygen consumption, lactate threshold, and running economy. However, other factors also determine success and progression of high-level endurance athletes. Therefore, the aim was to identify the relevant factors for endurance performance assessed by international experts by adhering to a structured communication method (i.e., Delphi technique). Three anonymous evaluation rounds were conducted initiated by a list of candidate factors (*n* = 120) serving as baseline input variables. The items that achieved ≥70% of agreement in round 1 were re-evaluated in a second round. Items with a level of agreement of ≥70% in round 2 reached consensus and items with a level of agreement of 40–69% in round 2 were re-rated in a third round followed by a consensus meeting. Round 1 comprised of 27 panellists (*n* = 24 male) and in round 2 and 3 18 (*n* = 15 male) of the 27 panellists remained. Thus, the final endurance expert panel comprised of 18 international experts (*n* = 15 male) with 20 years of experience on average. The consensus report identified the following 26 factors: endurance capacity, running economy, maximal oxygen consumption, recovery speed, carbohydrate metabolism, glycolysis capacity, lactate threshold, fat metabolism, number of erythrocytes, iron deficiency, muscle fibre type, mitochondrial biogenesis, hydrogen ion buffering, testosterone, erythropoietin, cortisol, hydration status, vitamin D deficiency, risk of non-functional overreaching and stress fracture, healing function of skeletal tissue, motivation, stress resistance, confidence, sleep quality, and fatigue. This study provides an expert-derived summary including 26 key factors for endurance performance, the “FENDLE” factors (FENDLE = Factors for ENDurance Level). This consensus report may assist to optimize sophisticated diagnostics, personalized training strategies and technology.

## Introduction

High performance in endurance sports is the result of the interplay of various factors including optimal training [1], recovery [2, 3], nutritional strategies [4–6], the use and handling of environmental [7] and psycho-social factors [8, 9] as well as high-tech equipment [10–12]. In addition, optimal biological factors are crucial to achieve world class competition level. For instance, recent scientific evidence suggests that a complex network of genetic and other biological mechanisms (e.g., transcriptomics, epigenomics, proteomics, metabolomics) contribute to the performance level of an athlete [13]. By integrating such novel research disciplines (omics) and advanced technology (e.g., machine learning) exercise science is shifting towards “precision exercise” offering training and recovery strategies tailored to the individual needs of an athlete [14–16]. The main idea of precision exercise is to base training and recovery decisions on individual characteristics including various biomarkers, ultimately attempting to personalize training programs, minimize injury risks, optimize performance outcomes, and to identify talents in near future [16]. These attempts, e.g., in the field of precision medicine, are based on prediction models and the predictive ability of these models tend to increase with the amount and quality of data input [17]. Thus, from a “precision endurance exercise” perspective, the more factors associated with high-level endurance performance are integrated, the better the prediction of the model should become as evidenced in medical research [18].

However, from a practical and a scientific point of view little agreement exists on the most important factors that influence high-level endurance performance. A first step towards developing precision exercise for endurance athletes would be to identify the relevant key factors for further modelling. In addition, endurance athletes, their coaches, and researchers could be informed and guided how to set priorities regarding training strategies and future research. For example, the identified key factors could be assessed with innovative wearable technology. Therefore, the aim of this study was to reach consensus about the key factors that are considered important for high-level endurance performance among international experts. The Delphi technique allows a comprehensive and structured group communication process aiming to achieve convergence of expert opinions by employing iterative data collection [19–24].

## Materials and methods

### Study design and consensus threshold

The study protocol is available at the open science framework (doi:10.17605/OSF.IO/YH5V4). We conducted a consensus study by employing the Delphi technique according to the description by Hsu *et al*., [19] the checklist by Sinha *et al*., [25] and Hasson *et al*., [26]. We also followed the recommendations for Conducting and REporting DElphi Studies (CREDES) [27]. The 12-item CREDES checklist is enclosed in S1 Table. The study was implemented in three phases: 1) preparation, 2) conduction, and 3) analysis [28]. The Delphi process (i.e., conduction phase) consisted of three iterative rounds of web-based questionnaires, participant response, and controlled feedback.

We employed a dichotomous scale (relevant vs. not relevant) to determine the relevance of the items. A cut-off value of 70% level of agreement was *a priori* chosen as the consensus threshold [27]. Level of agreement was categorized into low (0–39%), moderate (40–69%), and high (70–100%). Items with a low or moderate level of agreement were considered not relevant whereas items with a high level of agreement (≥70%) represented relevant factors and/or consensus. The steering committee advised on study as well as survey design, methodology, and content, but did not interact with or act on behalf of the panellists. Ethical approval for conducting the study was obtained from the Ethical Review Committee Health, Medicine, and Life Science of Maastricht University (FHML-REC/2019/021) and conducted in accordance with the Declaration of Helsinki for human research.

### Study flow

**Fig 1:**
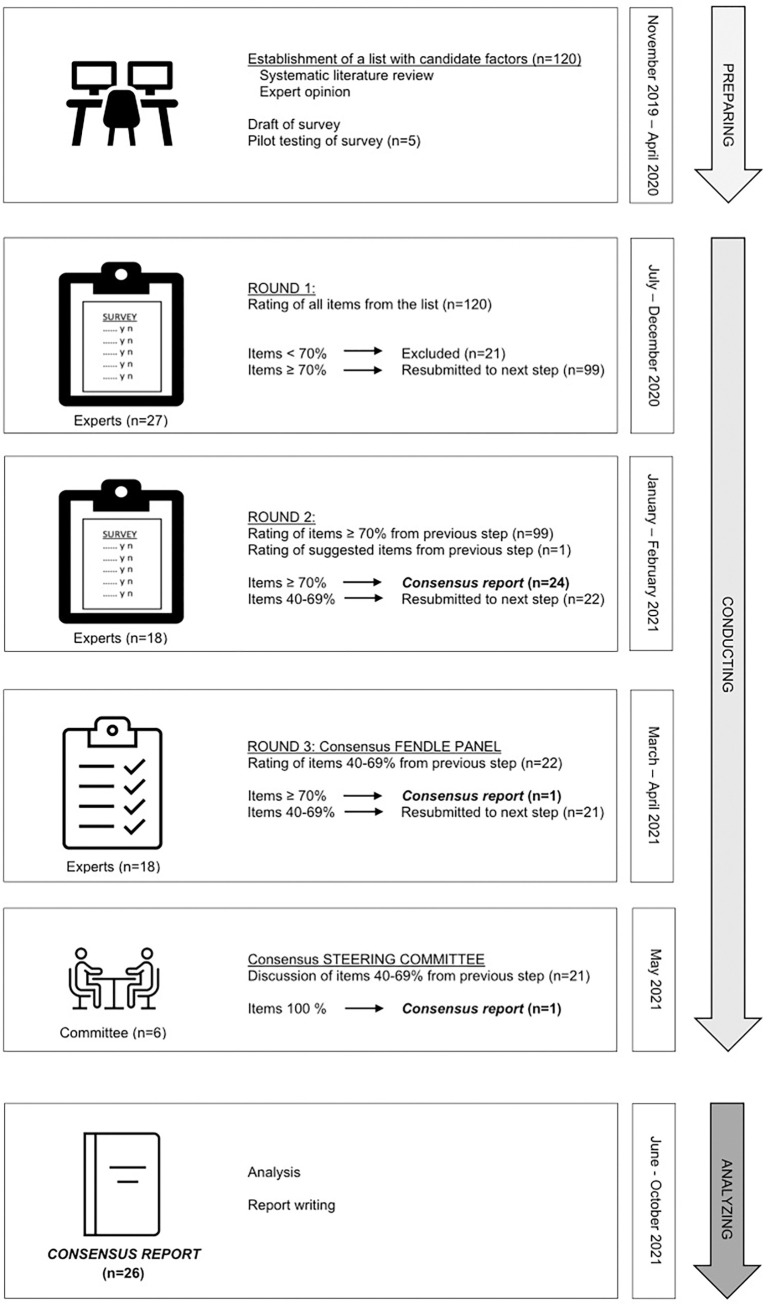
Study flow.

An initial list of candidate factors (*n* = 120) established by the steering committee served as input for the first round. Before distribution, the survey was pilot-tested and subsequently sent to the panellists of round 1. The items receiving a high level of agreement in round 1 were re-evaluated in a second round. All high level of agreement items in round 2 were included in the ‘consensus report’ presenting the level of agreement for each item. Moderate level of agreement items after round 2 were resubmitted to round 3 and in case the 70% threshold was attained, these factors were also added to the consensus report. Finally, a ‘consensus decision’ with the steering committee took place in which the remaining items of round 3 were considered for additional inclusion into the consensus report. The consensus report thus contains the factors that achieved a high level of agreement (i.e., the “FENDLE” factors). The study flow is illustrated in Fig 1.

### Panellists

In accordance with the CREDES recommendations [27] we involved experts with diverse backgrounds (e.g., high-level coaches, exercise scientists, physicians, etc.) and geographical locations. Inclusion criteria to participate in this study were: 1) age of 18 years or older; 2) good command of English language; 3) competence in or experience with (elite) endurance training. The panellists were recruited through non-probability purposive sampling to ensure that the invited experts represent sufficient experience, knowledge, and interest in the topic. Eligible panellists were professional athletes or coaches, exercise-scientists, physiotherapists, exercise-psychologists, or medical physicians. The panellists had to possess extensive experience and knowledge of elite endurance performance. The qualifications and responsibilities of the expert panel are displayed in S2 Table. Invitations to take part in this study were sent through email by the steering committee members using purposive sampling. The invitation letter included the participant information leaflet containing information regarding the aim of the study, the Delphi process, what was expected from the panellists, the projected timeline, and the amount of time, effort, and commitment required to finish the study. The panellists remained anonymous except for the main investigator (MK) for communication purposes. Each survey took approximately 15 minutes to complete.

### Data collection

The web-based surveys were conducted using Qualtrics (Qualtrics, Provo, UT). The panellists provided written informed consent for the study at the start of the first survey. In all three rounds of questionnaires, the panellists were asked to rate the proposed items as either ‘relevant’ or ‘not relevant’: “Which of the following factors, if any, are relevant for endurance performance?” Following each round the responses were analysed and anonymously reported back to the experts (e.g., number of panellists, level of agreement for each item) allowing them to reconsider pervious decisions.

### Preparation (PHASE I)

The purpose of the preparation phase was to develop a comprehensive list of candidate factors that are possibly associated with high-level endurance performance and could potentially be incorporated into the consensus report. First, a literature search was performed in the Medline database to identify candidate factors (last search date: 05. November 2019). All retrieved articles were read by the main investigator (MK). The potentially relevant factors were extracted using Excel and compiled into a list. Risk of bias of individual studies was not assessed. The list was subsequently revised (e.g., wording of factors, adding/ removing factors) based on recommendations from the steering committee. Finally, the list comprised of 120 factors (S3 Table) and served as input for the first round of the Delphi process [19]. Before distribution, the survey was pilot tested by five experts with varying backgrounds (exercise scientist, athletes, coaches) and 14 years of experience on average, different from those recruited for the main study, to verify the clarity of questions. The testers were instructed not to add or remove factors from the list. Suggestions from the testers were used to revise the survey.

### Conduction (PHASE II)

#### Round 1

For reason of efficiency and proposed by the pilot testers, the panellists were asked to ‘uncheck’ the non-relevant factors from the list in the first round. We also invited the experts to add missing factors and to provide free text comments. Finally, we requested relevant demographic data, such as name, age-group, email address, profession, place of employment, and quantifiable level of expertise.

#### Round 2

Based on the results from round 1, the steering committee agreed to eliminate the candidate factors that did not reach the threshold of 70%. Thus, items that did not reach the 70% threshold in round 1 were discarded from the survey and not resubmitted in round 2. In round 2, the items that attained a level of agreement of ≥70% in round 1 were distributed to the panellists who completed round 1 together with the average level of agreement, the newly proposed items, and the anonymized free text comments from round 1. This time, the panellists were asked to ‘check’ the relevant factors. After round 2, the items that achieved a level of agreement of ≥70% reached consensus and were incorporated in the consensus report.

### Round 3 and consensus decision of the steering committee

In round 3, we provided the anonymized results of the second survey to the experts who completed both rounds (i.e., the “FENDLE PANEL”) including their own rating and group summary statistics. In this round, the experts had the option to re-rate the items that reached a moderate level of agreement (40–69%) in round 2. In this ‘consensus decision’ of the FENDLE PANEL, the items that achieved a level of agreement of ≥70% after round 3 were also added to the consensus report. The remaining factors from round 3 were set aside for the ‘consensus decision’ amongst the steering committee members. Each member of the steering committee could propose items which were consequently discussed by the group. In case each member agreed (level of agreement = 100%), the proposed factor was added to the consensus report. Lastly, in round 2 and 3, there was no option for free text responses. S4 Table contains a link to the web-based questionnaire of round 1 and 2 as well as an illustration of round 3.

### Analysis (PHASE III)

After each round the raw data was downloaded from Qualtrics. We checked for incomplete submissions and duplicates. In case duplicate panellists were identified, the first survey submitted was analysed. Incomplete submissions were excluded. In case a valid email address was provided, the research team invited the participant to finish incomplete surveys. For the data analysis, we randomly assigned identification numbers to the experts so that they remained pseudonymous. Descriptive (frequency, percentage) and inferential statistics (mean, median, interquartile range) were used to describe the demographics and to analyse the extent of consensus [26]. The main outcome variable was binary. Secondary outcome variables were both categorical (gender, age-group, country, and occupation) and continuous (years of experience). All analysis were performed with R (version 4.0.3).

### Results

#### Survey distribution and demographics of panellists

Twenty-seven panellists completed the first round of questionnaire (July–December 2020) and were consequently invited to participate in round 2. Eighteen panellists (66.7%) from nine countries finished round 2 (January–February 2021) and 3 (March–April 2021) and thus completed all three rounds of questionnaires. Of the 18 panellists, 15 (83.3%) were male and 72.2% were aged between 31 and 60 years. The median time in years (interquartile range) of practical experience with endurance athletes was 20.0 (10.0–33.5). Table 1 demonstrates the demographics of the panellists. Finally, the consensus decision of the steering committee took place in May 2021.

**Table 1:** Demographics of the panellists.

	ROUND 1	ROUND 2 & 3
		i.e., FENDLE PANEL
Total (n, %)	27 (100)	18 (100)
Gender (n, %)		
Males	24 (88.9)	15 (83.3)
Females	2 (7.4)	2 (11.1)
Missing	1 (3.7)	1 (5.6)
Age group in years (n, %)		
18–30	5 (18.5)	4 (22.3)
31–60	20 (74.1)	13 (72.1)
60+	1 (3.7)	1 (5.6)
Missing	1 (3.7)	0
Country (n, %)		
Canada	1 (3.7)	1 (5.6)
USA	1 (3.7)	1 (5.6)
United Kingdom	2 (7.4)	1 (5.6)
Belgium	4 (14.8)	3 (16.7)
Germany	3 (11.1)	2 (11.1)
Italy	3 (11.1)	2 (11.1)
Netherlands	8 (29.7)	5 (27.6)
Norway	1 (3.7)	1 (5.6)
Sweden	2 (7.4)	2 (11.1)
Finland	1 (3.7)	0
Spain	1 (3.7)	0
Occupation (n, %)		
Chief Executive Officer (triathlon)	1 (3.7)	1 (5.6)
PhD student (sport science)	5 (18.5)	4 (22.2)
Physician (sports and rehabilitation)	2 (7.4)	1 (5.6)
Physiotherapist	1 (3.7)	1 (5.6)
Professor (sport science)	6 (22.3)	6 (33.1)
Director (triathlon)	1 (3.7)	1 (5.6)
Head Coach		
Road cycling	1 (3.7)	1 (5.6)
Soccer	1 (3.7)	1 (5.6)
Triathlon	2 (7.4)	2 (11.1)
Unknown	4 (14.8)	0
Missing	3 (11.1)	0
Practical experience in years (median, IQR^a^)	20.0 (9.0–33.0)	20.0 (10.0–33.5)

FENDLE: acronym for Factors for ENDurance Level.

^a^IQR = interquartile range.

### Survey results

Fig 1 illustrates the flow of the factors through the Delphi process. In the first round, 99 of the 120 candidate items achieved the 70% threshold and consequently were resubmitted in round 2. In addition, one item (sedentary lifestyle) was proposed as new factor and therefore added to round 2. In the second round, 24 of 100 items achieved consensus ≥70% and thus were included in the consensus report. Further, 22 items were rated with a moderate level of agreement and therefore resubmitted in round 3. During the ‘consensus decision’ of the FENDLE PANEL in round 3, one item “endurance capacity” was rated as relevant (level of agreement 72%) and hence integrated into the consensus report. S5–S7 Tables displays the results of round 1–3, respectively. After round 3, the ‘consensus decisions’ of the steering committee took place, in which 13 of the 21 remaining items from round 3 were subject for discussion. The steering committee agreed (100%) on one item, “recovery speed”, to be additionally included in the consensus report. S8 Table presents the results of the consensus decision of the steering committee. The final consensus report contained 26 factors (Table 2) considered relevant for high-level endurance training and/or performance. Lastly, 20 factors were rated with a moderate (S9 Table) and 54 with a low level of agreement, representing the factors rated as not relevant (S10 Table).

**Table 2:** Consensus report describing the FENDLE factors, the 26 factors considered the most important to influence endurance training and/or performance.

Cluster		Factor	Level of agreement (%)
Physiology	General	Endurance capacity^a^	72.2
		Economy of movement	88.9
		Maximal oxygen consumption	94.4
		Recovery speed^b^	66.7
	Metabolism	Carbohydrate metabolism	100.0
		Glycolysis capacity	100.0
		Lactate threshold	88.9
		Fat metabolism	88.9
	Blood	Number of red blood cells	100.0
		Iron deficiency	94.4
	Muscle	Muscle fibres—type 1 vs. type 2a/x	94.4
		Mitochondrial biogenesis	88.9
		Hydrogen ion buffering	88.9
	Hormones	Testosterone	94.4
		Erythropoietin	83.3
		Cortisol	77.8
Nutrition		Electrolyte balance/ hydration status	77.8
		Vitamin D deficiency	72.2
Injuries		Risk of non-functional overreaching	88.9
		Risk of stress fracture	77.8
		Healing function of skeletal muscle tissue	88.8
Psychology		Motivation capacity	94.4
		Stress resistance	88.9
		Self-confidence	72.2
Fatigue		Sleep quality	94.4
		Level of fatigue	77.8

FENDLE: acronym for Factors for ENDurance Level.

^a^Added after the consensus decision of the FENDLE PANEL (round 3).

^b^Added after the consensus decision of the steering committee.

## Discussion

Based on an international expert consensus process (i.e., Delphi technique) the aim of this study was to identify key factors that are considered important for high-level endurance performance. In total, 26 factors achieved consensus. The 26 FENDLE factors comprised of five different clusters: i) physiology ii) nutrition, iii) injuries, iv) psychological traits and v) fatigue.

### Physiology

Traditionally, three physiological factors have been identified explaining endurance performance: i) ‘maximum oxygen consumption’ (i.e., the maximal capacity to take up, transport, and utilize oxygen), ii) the ability to maintain high velocity without accumulating blood lactate (‘lactate threshold’), and iii) ‘running economy’ often expressed as the oxygen utilized while running at a given constant speed [29]. Since these three physiological factors have been extensively investigated and linked to elite endurance performance, it seems reasonable that the expert panel identified these factors as relevant for high-level of endurance performance. Interestingly, this consensus report also identified the ability to quickly recover during and after endurance events (‘recovery speed’) as important factor for high-level endurance performance. Replenishing energy storage efficiently and quickly reduces time between training and competition and simultaneously can prevent injuries when training again with full energy storages [30]. The fuelling of the different energy pathways is a key factor for many endurance sports [4–6] and since most endurance disciplines involve either long-duration tasks (e.g., marathon running) and/or high-intensity exercise (e.g., 800m running) it seems plausible that the experts reached consensus about ‘glycolytic capacity’, ‘hydrogen iron buffering’, as well as ‘fat and carbohydrate metabolism’ as key factors influencing endurance performance. Furthermore, the muscle fibre spectrum [31, 32] including the density and efficiency of mitochondria [33] have substantial impact on endurance performance. It is well known that ‘fibre type distribution’ and ‘mitochondrial biogenesis’ are important for increasing oxygen utilization for energy production stimulated by various training methods [34, 35]. Oxygen delivery and utilization are critical components for the energy turnover in the muscle cells [36]. Substantial evidence indicates that improving oxygen transportation by the augmentation of haemoglobin levels (e.g., through high-altitude training, medication, or blood transfusion) significantly improves endurance performance [37]. Thus, it seems reasonable that the experts rated the ‘number of red blood cells’ and ‘iron deficiency’ as key factors for high-level endurance performance. Finally, three hormones reached consensus as key physiological factors for endurance performance: ‘testosterone’, ‘erythropoietin’, and ‘cortisol’. Testosterone is known to stimulate muscle mass and to reduce body fat [38]. Erythropoietin induces erythropoiesis, the maturation and proliferation of oxygen-delivering red blood cells [39]. Finally, in the skeletal muscle the level of cortisol plays a fundamental role in regulating energy homeostasis [40]. During exercise, the high level of cortisol increases the availability of metabolic substrates, protects from immune cell activity, and maintains vascular integrity [41].

### Injury and nutrition

Good health is essential for achieving high training volumes necessary for maximal endurance performances. Since endurance athletes engage in numerous low- to high intensity trainings and thus are exposed to constant muscle and tissue damage, it seems reasonable that the experts identified the ‘healing function of skeletal muscle tissue’, ‘risk of stress fracture’, and ‘risk of non-functional overreaching’ as key factors for high-level endurance performance. Moreover, tissue repair is supported by various nutritional factors. For example, a low level of vitamin D has been associated with maladaptation of skeletal muscle and bone tissue [42]. Proper muscle renewal in response to exercise is required for optimal hypertrophic effects [43]. Therefore, it is plausible that ‘vitamin D deficiency’ has reached consensus in the present analysis. Further, ‘hydration status’ and ‘electrolyte balance’ were rated as key endurance factors by the panellists. The loss of body fluids during exercise is mostly due to sweating and the replacement of sodium loss is widely recommended [44]. The hydration of athletes tends to vary according to individual factors (e.g., thirst response, acclimation, gut training) and environmental factors (e.g., ambient temperature, provision of drinks at aid stations) as well as the type and intensity of exercise, making individualized fluid replacement strategies necessary for high-level endurance performance [5, 45].

### Psychology and fatigue

This report contains three psychological key factors relevant for high-level endurance performance: ‘motivation’, ‘stress resistance’, and ‘self-confidence’. Motivation is a key factor when describing (long-term) success in endurance athletes. Intrinsic motivation determines the high engagement in disciplined training and therefore is crucial for any athlete [46]. Professional athletes must be able to deal with stress during training and competitions [47]. In literature, self-confidence is one of the most cited factors thought to affect athletic performance [48, 49]. Finally, it is well known that engaging in extensive endurance training induce central and peripheral fatigue [50]. Therefore, the ‘sleep quality’ and ‘level of fatigue’ are not surprising to be identified as key factors influencing high-level endurance performance.

### Areas of disagreement

The number of relevant endurance factors declined from 99 factors in the first round to 24 factors in the second round. For instance, the volume of heart (85%) and lungs (78%) were rated as relevant in the first round but have been discarded after the second round (level of agreement round 2: 33% and 17%, respectively). In addition, although myoglobin plays an important role in the oxidative capacity of endurance trained runners [51], ‘myoglobin storage capacity’ was rated by 89% of the experts as relevant in round 1, but only by 33% in round 2. Furthermore, zinc (level of agreement: 85% round 1, 28% round 2), magnesium (level of agreement: 85% round 1; 39% round 2), vitamin C (level of agreement: 78% round 1; 22% round 2), and Vitamin E (level of agreement: 74% round 1; 11% round 2) deficiency were eliminated after round 2. Zinc and magnesium are essential trace elements and normal level of zinc are needed for proper immune system function [52]. However, there is no evidence of reduced performance in zinc deficient endurance athletes [53]. Magnesium supplementation, in turn, may enhance athletic performance in deficient athletes [54], although is not beneficial when magnesium status is normal [55]. Based on current scientific knowledge, antioxidants (level of agreement: 82% round 1; 22% round 2) including Vitamin C and E supplements, may not provide additional benefits for athletes. Finally, the ‘risk of upper respiratory tract infections’ achieved a level of agreement of 67% after round 3 and thus has not been included in the consensus report (threshold 70%). Except for injuries, upper respiratory symptoms are the most common medical presentation in endurance athletes [56]. All in all, the factors that did not make it on the consensus report remain subject for discussion and should not be overlooked by athletes and coaches.

### Research implications

This consensus report provides athletes, coaches, and exercise scientists a holistic overview of the key factors contributing to high-level endurance performance. Consequently, these factors need to be recognized and prioritized in the future. The 26 factors included in the consensus report demonstrate where to focus on in the future. For instance, the current results can be integrated into novel research disciplines such as -omics approaches. The aim of such approaches is the implementation of precision medicine in sports [14], which attempt to personalize performance by employing prediction models [16]. The predictive ability of such models tends to increase with the amount and quality of data input [17]. Hence, the identification of key factors and integration into future models the better the predictions will become [18]. Moreover, the current findings can be integrated into new technology such as wearables for data-informed decision-making enabling endurance athletes and coaches to monitor the athletes’ health, risk of injury, and performance in real time [57]. In near future, through rapid advances in technology, more opportunities will arise to integrate the FENDLE factors. Finally, replication studies are warranted to validate the current findings and update the consensus report.

### Strengths and limitations

We would like to highlight several strengths. i) First, we strictly followed the study protocol and reported the methodological considerations undertaken transparently. ii) In the preparation phase, a literature review was performed. The identified studies served as theoretical framework for establishing a comprehensive list. This list may not have captured all endurance factors, although only one new item was proposed from the expert panel. iii) Before distribution, the survey was pilot tested to assure comprehension of the survey [58]. iv) The steering committee consisted of international experts from the field of sport science who advised on study and survey design, methodology, and content. v) The authors assured geographic dispersion and anonymity of the panellists. Furthermore, the expert panel did not interact directly with each other so that social pressure was avoided [28]. The panellists had also the option to provide free text comments in round 1 and could reconsider initial ratings. vi) Finally, the response rates in round 2 (66%) and 3 (100%) were high [59]. Engaging 15–20 panellists is sufficient as long as the background of panellists is homogenous, and three rounds is appropriate for reaching consensus within a Delphi process [19].

We would like to acknowledge some limitations: i) Purposive sampling might have introduced selection bias [60] and personal perceptions of the experts could have influenced the results. ii) We are aware that the final expert selection is a limitation of the current study. We advise to recruit a higher number of experts from more diverse backgrounds/ disciplines (e.g., accredited registered sports dietitians) representing in-depth knowledge in each area. iii) We used different approaches to answer the questions in round 1 and 2. Hence we recommend a follow up study employing coherent methods throughout the Delphi process. iv) Some factors may be classified as “higher-level” (e.g., maximal oxygen consumption) or as “lower-level” factors (e.g., ‘metabolic’, ‘blood’ related factors) with the lower-level factors influencing endurance performance indirectly by affecting the higher-level factors. Hence, some potentially meaningful (and likely lower-level) factors may have been omitted (e.g., ‘strength/ power’; level of agreement 33.3%). v) Although experts from various disciplines were recruited, most factors identified pertain to running. Factors related to sport-specific skills, such as ‘technique in swimming’ or ‘power in cycling’ are underrepresented. vi) Factors such as ‘exogenous carbohydrate/ caffeine/ post exercise protein ingestion’, ‘neuromuscular control’, ‘strategy’ or ‘decision making’ were not examined in the current study, although these factors have extensively been reported and linked to endurance performance or recovery [5, 6, 61–64]. vii) Expert opinion remains among the lowest levels of empirical evidence [65] and the findings are only as valid as the opinions of the experts constituting the panel. Nonetheless, the experts of our study had on average 20 years of practical experience, reflecting a trained panel with good knowledge of the topic. viii) Finally, Delphi studies are considered evidence-based approaches with emphasis on value of expert judgement, which is not accessible through clinical trials [27]. This technique has the potential to arrive at valid and credible results—if performed properly. We therefore strictly followed the quality criteria for conducting and reporting Delphi studies to increase the validity of the results.

## Conclusions

This study provides an expert-derived consensus report identifying the important factors for high-level endurance performance, the FENDLE factors. We offer professional coaches, athletes, and scientist insights into 26 key endurance factors and strongly recommend considering these factors when optimizing personalized training strategies and technology in the future.

## Supporting information

S1 Table CREDES checklist. (PDF)

S2 Table Qualifications and responsibilities of the expert panel. (PDF)

S3 Table List of candidate factors (n = 120). (PDF)

S4 Table Survey outline. (PDF)

S5 Table Results of round 1. (PDF)

S6 Table Results of round 2. (PDF)

S7 Table Results of round 3. (PDF)

S8 Table Consensus decision. (PDF)

S9 Table Moderate level of agreement factors. (PDF)

S10 Table Low level of agreement factors. (PDF)
