# Supplementary material for: Factors associated with high-level endurance performance: An expert consensus derived via the Delphi technique
Source: PLoS One. 2022 Dec 27;17(12):e0279492. doi: 10.1371/journal.pone.0279492 (PMC9794057; doi:10.1371/journal.pone.0279492)
Supplement: S2 Table — (PDF) [file pone.0279492.s002.pdf]

S2 Table. Qualifications and responsibilities of the expert panel.

Qualifications<br>- Possess extensive experience with and knowledge of endurance performance<br>- Interest in the topic
Responsibilities<br>- Participate anonymously in three rounds of questionnaires (15 minutes each)
