# Supplementary material for: Factors associated with high-level endurance performance: An expert consensus derived via the Delphi technique
Source: PLoS One. 2022 Dec 27;17(12):e0279492. doi: 10.1371/journal.pone.0279492 (PMC9794057; doi:10.1371/journal.pone.0279492)
Supplement: S3 Table — (PDF) [file pone.0279492.s003.pdf]

S3 Table. List of candidate factors (*n*=120).

1. **<u>Training factors:</u>**
   Endurance capacity
   Maximal oxygen consumption
   Economy of movement (=energy utilization)
   Strength capacity
   Power capacity
   Speed capacity
   Lactate threshold
   Lung volume
   Heart volume
   Coordination capacity
   Flexibility capacity
   Agility capacity
   Reaction time
   Recovery speed

2. **<u>Metabolism factors:</u>**
   Basal metabolism rate (=calories required to keep the body functioning at rest)
   Glycolysis capacity (=break down of glucose)
   Mitochondrial biogenesis (=growth of pre-existing mitochondria)
   Myoglobin storage capacity (=iron/ oxygen-binding protein)
   Thermogenesis (=production of heat in the body)
   Angiogenesis (=formation of new blood vessels)
   Fat metabolism (break down of fat for energy)
   Creatine kinase metabolism
   Lactate dehydrogenase metabolism
   Lactate buffering system (=regulation of lactate level)

3. **<u>Body factors:</u>**
   Weight / BMI
   Total fat mass
   Regional fat mass
   Subcutaneous adipose tissue (=fat under the skin)
   Visceral adipose tissue (=fat around internal organs)
   Lean mass (=mass of all organs except body fat including bones, muscles, blood, skin)
   Bone mineral density
   Tendon stiffness
   Number of red blood cells (=erythrocytes)
   Muscle fibres - hypertrophy capacity (=muscle growth)
   Muscle fibres - type 1 vs. type 2a/b (=slow vs. fast twitch fibres)
   Muscle fibres - transformation capacity (type 1 vs. type 2)
   Muscle fibres - contraction velocity capacity

4. **<u>Hormone metabolism:</u>**

Erythropoietin (EPO) level
Insulin-like growth factor-1 (IGF-1) level
Growth hormone level
Cortisol level
Epinephrine level
Norepinephrine level
Testosterone level
Dihydrotestosterone level
Oestradiol level
Dehydroepiandrosterone level
Ghrelin level
Progesterone level
Follicle-stimulating hormone level
Gonadocorticoids level
Human chorionic gonadotropin level
Gonadotropin-releasing hormone level
Thyroid hormones level
Androstenedione level
Anti-Müllerian hormone level

5. **<u>Nutrition metabolism:</u>**

Valine level
Leucine level
L-carnitine level
Carnosine level
Creatine level
Carbohydrate metabolism
Saturated fat metabolism
Unsaturated fat metabolism
Cholesterol level
Omega 3 level
Omega 6 level

<u>Vitamin deficiencies</u>

Vitamin A deficiency
Beta carotene deficiency
Vitamin B complex vitamins (B1-12) deficiency
Vitamin C deficiency
Vitamin D deficiency
Vitamin E deficiency
Vitamin K deficiency
Folic acid deficiency

<u>Mineral deficiencies</u>

Iron deficiency
Zinc deficiency
Magnesium deficiency
Selenium deficiency
Gluten intolerance
Lactose intolerance
Caffeine metabolism
Alcohol metabolism
Antioxidant level

Bicarbonate level
Cell hydration status
Electrolyte balance/ hydration status
Steroid metabolism

6. **Immune system:**
   Detoxification process
   Cytokine responses
   Healing function of skeletal tissue
   Healing function of soft tissue
   Blood pressure regulation

7. **Injuries:**
   Risk of left ventricular hypertrophy
   Risk of metabolic myopathy
   Risk of stress fractures
   Risk of upper respiratory tract infections
   Risk of non-functional overreaching
   Risk of joint injuries
   Risk of lumbar disk degeneration
   Risk of inguinal hernia

8. **Psychological factors:**
   Stress resistance
   Motivation capacity
   Resilience capacity
   Concentration capacity
   Emotion regulation
   Pain sensitivity
   Aggression regulation
   Self-control
   Self-confidence
   Risk of eating disorders
   Risk of addiction
   Intro vs. extroverted personality
   Ability to differentiate

9. **Environmental factors:**
   Smoking behaviour
   Alcohol usage
   Sleep quality
   Level of fatigue
   Heat resistance capacity
   Altitude training sensitivity
