# Supplementary material for: Factors associated with high-level endurance performance: An expert consensus derived via the Delphi technique
Source: PLoS One. 2022 Dec 27;17(12):e0279492. doi: 10.1371/journal.pone.0279492 (PMC9794057; doi:10.1371/journal.pone.0279492)
Supplement: S4 Table — (PDF) [file pone.0279492.s004.pdf]

S4 Table. Survey outline.

Delphi Study - round 1 (click on link to access survey)

Delphi Study - round 2 (click on link to access survey)

Illustration of round 3

Factor	Level of agreement (%)	Rating round 2	Rating round 3
Endurance capacity	61,1	N	
Recovery speed	61,1	N	
Angiogenesis (=formation of new blood vessels)	50,0	Y	
Muscle fibres - transformation capacity (type 1 vs. type 2)	55,6	N	
Weight / BMI	44,4	N	
Total fat mass	50,0	Y	
Lean mass (=mass of all organs except body fat including bones, muscles, blood, skin)	44,4	Y	
Tendon stiffness	55,6	N	
Insulin-like growth factor-1 (IGF-1) level	55,6	N	
Growth hormone level	66,7	Y	
Vitamin B complex vitamins (B1-12) deficiency	50,0	N	
Blood pressure regulation	50,0	N	
Healing function of soft tissue	50,0	N	
Risk of joint injuries	66,7	Y	
Risk of upper respiratory tract infections	61,1	N	
Emotion regulation	66,7	N	
Pain sensitivity	44,4	Y	
Self-control	50,0	N	
Resilience capacity	50,0	Y	

Concentration capacity	44,4	Y	
Heat resistance capacity	50,0	Y	
Altitude training sensitivity	55,6	N	

Y=Yes (Factor is relevant and should be included in the consensus report).

N=No (Factor is not relevant and should not be included in the consensus report).
