# Supplementary material for: Factors associated with high-level endurance performance: An expert consensus derived via the Delphi technique
Source: PLoS One. 2022 Dec 27;17(12):e0279492. doi: 10.1371/journal.pone.0279492 (PMC9794057; doi:10.1371/journal.pone.0279492)
Supplement: S5 Table — (PDF) [file pone.0279492.s005.pdf]

S5 Table. Results of round 1.

Table A. Factors rated as ‘relevant’ in round 1 (level of agreement 70-100%), *n*=99.

	Factor	Level of agreement (%)
Training	Endurance capacity	96,3
	Maximal oxygen consumption	100,0
	Economy of movement (=energy utilization)	96,3
	Strength capacity	70,4
	Power capacity	85,2
	Lactate threshold	96,3
	Lung volume	77,8
	Heart volume	85,2
	Recovery speed	88,9
Metabolism	Glycolysis capacity (=break down of glucose)	100,0
	Mitochondrial biogenesis (=growth of pre-existing mitochondria)	100,0
	Myoglobin storage capacity (=iron/ oxygen-binding protein)	88,9
	Thermogenesis (=production of heat in the body)	70,4
	Angiogenesis (=formation of new blood vessels)	85,2
	Fat metabolism (break down of fat for energy)	88,9
	Lactate dehydrogenase metabolism	85,2
	Lactate buffering system (=regulation of lactate level)	96,3
Body	Weight / BMI	88,9
	Total fat mass	88,9
	Subcutaneous adipose tissue (=fat under the skin)	70,4
	Lean mass (=mass of all organs except body fat including bones, muscles, blood, skin)	88,9
	Tendon stiffness	88,9
	Number of red blood cells (=erythrocytes)	100,0
	Muscle fibres - hypertrophy capacity (=muscle growth)	70,4
	Muscle fibres - type 1 vs. type 2a/b (=slow vs. fast twitch fibres)	100,0
	Muscle fibres - transformation capacity (type 1 vs. type 2)	92,6
	Muscle fibres - contraction velocity capacity	74,1
Hormones	Erythropoietin (EPO) level	92,6
	Insulin-like growth factor-1 (IGF-1) level	92,6

	Growth hormone level	92,6
	Cortisol level	96,3
	Epinephrine level	77,8
	Norepinephrine level	77,8
	Testosterone level	100,0
	Dihydrotestosterone level	88,9
	Oestradiol level	85,2
	Dehydroepiandrosterone level	70,4
	Ghrelin level	74,1
	Progesterone level	77,8
	Follicle-stimulating hormone level	70,4
	Gonadocorticoids level	77,8
	Human chorionic gonadotropin level	70,4
	Gonadotropin-releasing hormone level	77,8
	Thyroid hormones level	81,5
	Androstenedione level	77,8
Nutrition	Valine level	70,4
	Leucine level	85,2
	L-carnitine level	81,5
	Carnosine level	77,8
	Creatine level	81,5
	Carbohydrate metabolism	100,0
	Saturated fat metabolism	77,8
	Unsaturated fat metabolism	74,1
	Cholesterol level	74,1
	Omega 3 level	74,1
	Omega 6 level	70,4
	Vitamin A deficiency	74,1
	Beta carotene deficiency	77,8
	Vitamin B complex vitamins (B1-12) deficiency	88,9
	Vitamin C deficiency	77,8
	Vitamin D deficiency	92,6
	Vitamin E deficiency	74,1
	Folic acid deficiency	77,8
	Iron deficiency	100,0
	Zinc deficiency	85,2
	Magnesium deficiency	85,2
	Selenium deficiency	74,1
	Caffeine metabolism	81,5

	Antioxidant level	81,5
	Bicarbonate level	77,8
	Cell hydration status	88,9
	Electrolyte balance/ hydration status	96,3
	Steroid metabolism	92,6
Immune system	Detoxification process	81,5
	Cytokine responses	85,2
	Healing function of skeletal tissue	88,9
	Healing function of soft tissue	81,5
	Blood pressure regulation	85,2
Injuries	Risk of left ventricular hypertrophy	74,1
	Risk of metabolic myopathy	70,4
	Risk of stress fractures	85,2
	Risk of upper respiratory tract infections	85,2
	Risk of non-functional overreaching	88,9
	Risk of joint injuries	88,9
Psychological	Stress resistance	100,0
	Motivation capacity	100,0
	Resilience capacity	92,6
	Concentration capacity	92,6
	Emotion regulation	96,3
	Pain sensitivity	96,3
	Self-control	96,3
	Self-confidence	100,0
	Risk of eating disorders	85,2
Environment	Smoking behaviour	74,1
	Alcohol usage	85,2
	Sleep quality	96,3
	Level of fatigue	96,3
	Heat resistance capacity	85,2
	Altitude training sensitivity	85,2

Table B. Factors rated as 'moderate' in round 1 (level of agreement 40-69%), *n*=19.

	Factor	Level of agreement (%)
Training	Speed capacity	51,9
	Coordination capacity	63,0
	Flexibility capacity	59,3
Metabolism	Basal metabolism rate (=calories required to keep the body functioning at rest)	59,3
	Creatine kinase metabolism	55,6
Body	Regional fat mass	66,7
	Visceral adipose tissue (=fat around internal organs)	55,6
	Bone mineral density	51,9
Hormones	Anti-Müllerian hormone level	55,6
Nutrition	Vitamin K deficiency	66,7
	Gluten intolerance	55,6
	Lactose intolerance	59,3
	Alcohol metabolism	48,1
Injuries	Injuries Risk of lumbar disk degeneration	59,3
	Injuries Risk of inguinal hernia	55,6
Psychological	Aggression regulation	66,7
	Risk of addiction	66,7
	Intro vs. extroverted personality	59,3
	Ability to differentiate	66,7

Table C. Factors rated as 'not relevant' in round 1 (level of agreement 0-39%), *n*=2.

	**Factor**	**Level of agreement (%)**
Training	Agility capacity	25,9
	Reaction time	14,8

Table D. Proposed factors from round 1.

(Sedentary) lifestyle in amateur athletes

Table E. Free text comments from round 1.

"For my studies, human muscle fibers are classified in 1, 2a and 2x. 2b fibers are present only in some animals, but not in human. Feel free to accept or not my suggestion, just a thought."
"List is complete"
"Since I'm not an expert in hormonal function, my opinion of these factors might not be very accurate."
"Without incorporating a number of people, it would have taken some time to come to this list. On the opposite end, the list is very comprehensive, or is it just very long."
