# Supplementary material for: Factors associated with high-level endurance performance: An expert consensus derived via the Delphi technique
Source: PLoS One. 2022 Dec 27;17(12):e0279492. doi: 10.1371/journal.pone.0279492 (PMC9794057; doi:10.1371/journal.pone.0279492)
Supplement: S6 Table — (PDF) [file pone.0279492.s006.pdf]

S6 Table. Results of round 2.

Table A. Factors rated as 'relevant' in round 2 (level of agreement 70-100%), *n*=24.

	**Factor**	**Level of agreement (%)**
Training	Maximal oxygen consumption	94,4
	Economy of movement (=energy utilization)	88,9
	Lactate threshold	88,9
Metabolism	Glycolysis capacity (=break down of glucose)	100,0
	Mitochondrial biogenesis (=growth of pre-existing mitochondria)	88,9
	Lactate buffering system (=regulation of lactate level)	88,9
	Fat metabolism (break down of fat for energy)	88,9
Body	Number of red blood cells (=erythrocytes)	100,0
	Muscle fibres - type 1 vs. type 2a/x (=slow vs. fast twitch fibres)	94,4
Hormones	Testosterone level	94,4
	Cortisol level	77,8
	Erythropoietin (EPO) level	83,3
Nutrition	Carbohydrate metabolism	100,0
	Iron deficiency	94,4
	Electrolyte balance/ hydration status	77,8
	Vitamin D deficiency	72,2
Immune system	Healing function of skeletal tissue	88,9
Injuries	Risk of non-functional overreaching	88,9
	Risk of stress fractures	77,8
Psychological	Stress resistance	88,9
	Motivation capacity	94,4
	Self-confidence	72,2
Environment	Sleep quality	94,4
	Level of fatigue	77,8

Table B. Factors rated as ‘moderate’ in round 2 (level of agreement 40-69%), *n*=22.

	**Factor**	**Level of agreement (%)**
Training	Endurance capacity	61,1
	Recovery speed	61,1
Metabolism	Angiogenesis (=formation of new blood vessels)	50,0
Body	Muscle fibres - transformation capacity (type 1 vs. type 2)	55,6
	Weight / BMI	44,4
	Total fat mass	50,0
	Lean mass (=mass of all organs except body fat including bones, muscles, blood, skin)	44,4
	Tendon stiffness	55,6
Hormones	Insulin-like growth factor-1 (IGF-1) level	55,6
	Growth hormone level	66,7
Nutrition	Vitamin B complex vitamins (B1-12) deficiency	50,0
Immune system	Blood pressure regulation	50,0
	Healing function of soft tissue	50,0
Injuries	Risk of joint injuries	66,7
	Risk of upper respiratory tract infections	61,1
Psychological	Emotion regulation	66,7
	Pain sensitivity	44,4
	Self-control	50,0
	Resilience capacity	50,0
	Concentration capacity	44,4
Environment	Heat resistance capacity	50,0
	Altitude training sensitivity	55,6

Table C. Factors rated as ‘not relevant’ in round 2 (level of agreement 0-39%), *n*=54.

	**Factor**	**Level of agreement (%)**
Training	Power capacity	33,3
	Heart volume	33,3
	Lung volume	16,7
	Strength capacity	16,7
Metabolism	Myoglobin storage capacity (=iron/ oxygen-binding protein)	33,3
	Lactate dehydrogenase metabolism	33,3
	Thermogenesis (=production of heat in the body)	5,6
Body	Muscle fibres – contraction velocity capacity	11,1
	Subcutaneous adipose tissue (=fat under the skin)	16,7
	Muscle fibres – hypertrophy capacity (=muscle growth)	11,1
Hormones	Dihydrotestosterone level	11,1
	Oestradiol level	33,3
	Thyroid hormones level	27,8
	Epinephrine level	11,1
	Norepinephrine level	11,1
	Progesterone level	11,1
	Gonadocorticoids level	11,1
	Gonadotropin-releasing hormone level	22,2
	Androstenedione level	11,1
	Ghrelin level	5,6
	Dehydroepiandrosterone level	5,6
	Follicle-stimulating hormone level	11,1
	Human chorionic gonadotropin level	5,6
Nutrition	Steroid metabolism	33,3
	Cell hydration status	33,3
	Leucine level	22,2
	Zinc deficiency	27,8
	Magnesium deficiency	38,9
	L-carnitine level	5,6
	Creatine level	22,2
	Caffeine metabolism	33,3
	Antioxidant level	22,2
	Carnosine level	16,7
	Saturated fat metabolism	11,1
	Beta carotene deficiency	11,1

	Vitamin C deficiency	22,2
	Folic acid deficiency	16,7
	Bicarbonate level	27,8
	Unsaturated fat metabolism	16,7
	Cholesterol level	22,2
	Omega 3 level	16,7
	Vitamin A deficiency	11,1
	Vitamin E deficiency	11,1
	Selenium deficiency	11,1
	Valine level	5,6
	Omega 6 level	11,1
Immune system	Cytokine responses	27,8
	Detoxification process	11,1
Injuries	Risk of left ventricular hypertrophy	27,8
	Risk of metabolic myopathy	11,1
Psychological	Risk of eating disorders	16,7
Environment	Alcohol usage	22,2
	Smoking behaviour	11,1
Proposed item	(Sedentary) lifestyle in amateur athletes	16,7
