# Supplementary material for: Factors associated with high-level endurance performance: An expert consensus derived via the Delphi technique
Source: PLoS One. 2022 Dec 27;17(12):e0279492. doi: 10.1371/journal.pone.0279492 (PMC9794057; doi:10.1371/journal.pone.0279492)
Supplement: S7 Table — (PDF) [file pone.0279492.s007.pdf]

S7 Table. Results of round 3.

Factors rated in round 3; *n*=22.

	**Factor**	**Level of agreement (%)**
Training	Endurance capacity[a,b]	72,2
	Recovery speed[†]	66,7
Metabolism	Angiogenesis (=formation of new blood vessels)[b]	55,6
Body	Muscle fibres - transformation capacity (type 1 vs. type 2)	55,6
	Weight / BMI	44,4
	Total fat mass	50,0
	Lean mass (=mass of all organs except body fat including bones, muscles, blood, skin)	44,4
	Tendon stiffness	55,6
Hormones	Insulin-like growth factor-1 (IGF-1) level	55,6
	Growth hormone level	66,7
Nutrition	Vitamin B complex vitamins (B1-12) deficiency[b]	55,6
Immune system	Blood pressure regulation	50,0
	Healing function of soft tissue	50,0
Injuries	Risk of joint injuries	66,7
	Risk of upper respiratory tract infections[b]	66,7
Psychological	Emotion regulation	66,7
	Pain sensitivity[b]	50,0
	Self-control	50,0
	Resilience capacity	50,0
	Concentration capacity	44,4
Environment	Heat resistance capacity	50,0
	Altitude training sensitivity	55,6

[a]Level of agreement achieved 70% threshold and therefore was included in the consensus report.

[b]Level of agreement changed compared to round 2.
