# Supplementary material for: Factors associated with high-level endurance performance: An expert consensus derived via the Delphi technique
Source: PLoS One. 2022 Dec 27;17(12):e0279492. doi: 10.1371/journal.pone.0279492 (PMC9794057; doi:10.1371/journal.pone.0279492)
Supplement: S8 Table — (PDF) [file pone.0279492.s008.pdf]

S8 Table. Consensus decision.

Results of the consensus decision of the steering committee, sorted by level of agreement.

	**Member 1**	**Member 2**	**Member 3**	**Member 4**	**Member 5**	**Level of agreement (%)**
Recovery speed[a]	Yes	Yes	Yes	Yes	Yes	100
Weight/ BMI	No	Yes	Yes	No	Yes	60
Tendon stiffness	Yes	No	Yes	No	Yes	60
Heat resistance capacity	Yes	Yes	No	Yes	No	60
Altitude training sensitivity	Yes	Yes	No	Yes	No	60
Angiogenesis	No	Yes	No	Yes	No	40
Muscle fibre transformation capacity	No	Yes	No	Yes	No	40
Healing function of soft tissue	No	Yes	No	No	No	20
Risk of joint injuries	No	Yes	No	No	No	20
Risk of upper respiratory tract infections	No	Yes	No	No	No	20
Emotion regulation	No	No	No	Yes	No	20
Self-control	No	No	No	Yes	No	20
Resilience	No	Yes	No	No	No	20

[a]100% level of agreement and the factor therefore was included in the consensus report.

Yes = Factor should be included in consensus report.

No = Factor should not be included in consensus report.
