# Supplementary material for: Factors associated with high-level endurance performance: An expert consensus derived via the Delphi technique
Source: PLoS One. 2022 Dec 27;17(12):e0279492. doi: 10.1371/journal.pone.0279492 (PMC9794057; doi:10.1371/journal.pone.0279492)
Supplement: S9 Table — (PDF) [file pone.0279492.s009.pdf]

S9 Table. Moderate level of agreement factors.

Factors that achieved a level of agreement of 40-69% after all three rounds (*n*=20).

	Factor	Level of agreement (%)
Metabolism	Angiogenesis	55,6
Body	Muscle fibre transformation capacity	55,6
	Tendon stiffness	55,6
	Total fat mass	50,0
	Weight / BMI	44,4
	Lean mass	44,4
Hormones	Growth hormone level	66,7
	Insulin-like growth factor-1 (IGF-1) level	55,6
Nutrition	Vitamin B complex vitamins (B1-12) deficiency	55,6
Immune system	Blood pressure regulation	50,0
	Healing function of soft tissue	50,0
Injuries	Risk of joint injuries	66,7
	Risk of upper respiratory tract infections	66,7
Psychological	Emotion regulation	66,7
	Pain sensitivity	50,0
	Self-control	50,0
	Resilience capacity	50,0
	Concentration capacity	44,4
Environment	Altitude training sensitivity	55,6
	Heat resistance capacity	50,0
