# Supplementary material for: Factors associated with high-level endurance performance: An expert consensus derived via the Delphi technique
Source: PLoS One. 2022 Dec 27;17(12):e0279492. doi: 10.1371/journal.pone.0279492 (PMC9794057; doi:10.1371/journal.pone.0279492)
Supplement: S10 Table — (PDF) [file pone.0279492.s010.pdf]

S10 Table. Low level of agreement factors.

Factors that achieved a level of agreement of 0-39% after all three rounds (*n*=54).

	Factor	Level of agreement (%)
Training	Power capacity	33,3
	Heart volume	33,3
	Lung volume	16,7
	Strength capacity	16,7
Metabolism	Myoglobin storage capacity	33,3
	Lactate dehydrogenase metabolism	33,3
	Thermogenesis	5,6
Body	Subcutaneous adipose tissue	16,7
	Muscle fibres - contraction velocity capacity	11,1
	Muscle fibres - hypertrophy capacity	11,1
Hormones	Oestradiol level	33,3
	Thyroid hormones level	27,8
	Gonadotropin-releasing hormone level	22,2
	Dihydrotestosterone level	11,1
	Epinephrine level	11,1
	Norepinephrine level	11,1
	Progesterone level	11,1
	Gonad corticoids level	11,1
	Androstenedione level	11,1
	Follicle-stimulating hormone level	11,1
	Ghrelin level	5,6
	Dehydroepiandrosterone level	5,6
	Human chorionic gonadotropin level	5,6
Nutrition	Magnesium deficiency	38,9
	Steroid metabolism	33,3
	Cell hydration status	33,3
	Caffeine metabolism	33,3
	Zinc deficiency	27,8
	Bicarbonate level	27,8
	Leucine level	22,2
	Creatine level	22,2
	Antioxidant level	22,2
	Vitamin C deficiency	22,2
	Cholesterol level	22,2

	Carnosine level	16,7
	Folic acid deficiency	16,7
	Unsaturated fat metabolism	16,7
	Omega 3 level	16,7
	Saturated fat metabolism	11,1
	Beta carotene deficiency	11,1
	Vitamin A deficiency	11,1
	Vitamin E deficiency	11,1
	Selenium deficiency	11,1
	Omega 6 level	11,1
	L-carnitine level	5,6
	Valine level	5,6
Immune system	Cytokine responses	27,8
	Detoxification process	11,1
Injuries	Risk of left ventricular hypertrophy	27,8
	Risk of metabolic myopathy	11,1
Psychological	Risk of eating disorders	16,7
Environment	Alcohol usage	22,2
	Smoking behaviour	11,1
Proposed factor	(Sedentary) lifestyle	16,7
